# First Molecular Survey and Genetic Characterization of *Rickettsia* spp. in *Haemaphysalis hystricis* Ticks Infesting Dogs in Taiwan

**DOI:** 10.3390/microorganisms13020424

**Published:** 2025-02-15

**Authors:** Chien-Ming Shih, Xing-Ru Huang, Esmeralda Erazo, Li-Lian Chao

**Affiliations:** 1 Graduate Institute of Medicine, College of Medicine, Kaohsiung Medical University, Kaohsiung 80708, Taiwan; cmshih@kmu.edu.tw (C.-M.S.);; 2 M.Sc. Program in Tropical Medicine, College of Medicine, Kaohsiung Medical University, Kaohsiung 80708, Taiwan; 3 Department of Medical Research, Kaohsiung Medical University Hospital, Kaohsiung 80708, Taiwan; 4 Center for Tropical Medicine and Infectious Disease Research, Kaohsiung Medical University, Kaohsiung 80708, Taiwan

**Keywords:** *Haemaphysalis hystricis*, tick, *Rickettsia* spp., Taiwan

## Abstract

*Rickettsia* infection in *Haemaphysalis hystricis* ticks infesting dogs was first screened in Taiwan by nested-PCR assay targeting the citrate synthase gene (*gltA*) of *Rickettsia*. A general infection rate (3.46%) was detected in a total of 1186 examined ticks, and infection rates of 3.20%, 3.6%, and 4.27% were detected in females, males, and nymphs, respectively. The monthly prevalence of *Rickettsia* infection was observed from March to November, and the highest infection was detected in April (6.92%) followed by a higher infection in July (5.56%), October (4.72%), September (3.57%), and May (3.54%). The prevalence of *Rickettsia* infection in ticks infesting stray dogs (4.15%) is significantly higher than ticks infesting domestic dogs (1.11%) (chi-square test, *p* = 0.015). Genetic analysis based on the *gltA* gene sequences from 13 Taiwan specimens, compared with 13 genospecies of *Rickettsia* strains documented in GenBank, revealed that the genetic identities of these Taiwan strains were phylogenetically affiliated with the genospecies of the transitional group (*R. felis*) and the spotted fever group (*R. aeschlimannii* and *R. raoultii*) of *Rickettsia*. This study demonstrates the first molecular screening of *Rickettsia* spp. in *H. hystricis* ticks infesting dogs in Taiwan. The human pathogenic strain of *R. aeschlimannii* was first discovered in *H. hystricis* ticks infesting dogs. Because dogs serve as companion animals to humans, the presence of various *Rickettsia* species existing in *H. hystricis* ticks may pose a potential threat to human health in Taiwan.

## 1. Introduction

Ticks are bloodsucking ectoparasitic arachnids which are commonly observed on canine hosts around the world [1,2]. The *Haemaphysalis hystricis* tick has been recorded in various countries of Southeast Asia including India, Vietnam, Myanmar, Thailand, Laos, Indonesia, Japan, China, and Taiwan [3]. Infested hosts of the *H. hystricis* tick have been recorded on pangolin, goat, cattle, wild boar, and dogs [4,5,6,7,8]. In our previous report, the *H. hystricis* tick was described as the second-most dominant tick species infesting dogs in Taiwan [9]. In addition, this tick species has been detected with various tick-borne pathogens including *Borrelia* and *Coxiella* in Malaysia [10,11], *Rickettsia* in China [6], and *Babesia* in Taiwan [5,12]. Although the *H. hystricis* tick was suspected as a potential vector tick for various tick-borne pathogens, a molecular survey regarding the existence and monthly prevalence of *Rickettsia* infection in *H. hystricis* ticks infesting dogs has never been conducted in Taiwan.

The *Rickettsia* microorganism is an obligate intracellular bacterium, and four major groups (i.e., ancestral group, typhus group, transitional group, and spotted fever group) have been identified from various hosts [13,14,15]. Tick-borne rickettsial infections have been recognized as a global threat of emerging and re-emerging tick-borne diseases for humans [16,17]. The hard ticks of *Ixodidae* have been recognized as the major vector for transmitting *Rickettsia* agents and have served as reservoir hosts for amplifying *Rickettsia* agents [18]. Indeed, transstadial and transovarial transmission of spotted fever group (SFG) rickettsiae have been described in various species of hard ticks [19,20]. Although most SFG rickettsiae are discovered in a particular geographic location [21,22,23,24,25,26], many SFG rickettsial species have been identified from Central and South America, Australia, and Asia [27,28,29,30,31,32,33]. However, genetic identification of rickettsial agents detected in *H. hystricis* ticks has never been reported in Taiwan.

Molecular analysis based on the citrate synthase gene (*gltA*) has been proved to be a useful tool for the phylogenetic analysis of the genetic diversity of *Rickettsia* species [34]. Indeed, previous investigations based on the molecular marker of the *gltA* gene have provided reliable information for analyzing the genetic relatedness among the diversity of *Rickettsia* species isolated from various ticks [35,36,37]. Therefore, molecular screening for *Rickettsia* infection and genetic identification of *Rickettsia* species based on the phylogenetic analysis of the *gltA* gene have made it feasible to discriminate the *Rickettsia* species within ticks.

The objectives of the present study are to determine the monthly prevalence of *Rickettsia* infection in *H. hystricis* ticks infesting dogs in Taiwan and to identify the genetic affiliation of *Rickettsia* agents detected in these ticks. Phylogenetic analysis of *Rickettsia* strains detected in *H. hystricis* ticks from Taiwan was compared with other *Rickettsia* strains documented in GenBank that have been validated from various biological and geographical origins (Table 1).

## 2. Materials and Methods

### 2.1. Tick Collection and Species Identification

**Figure 1:**
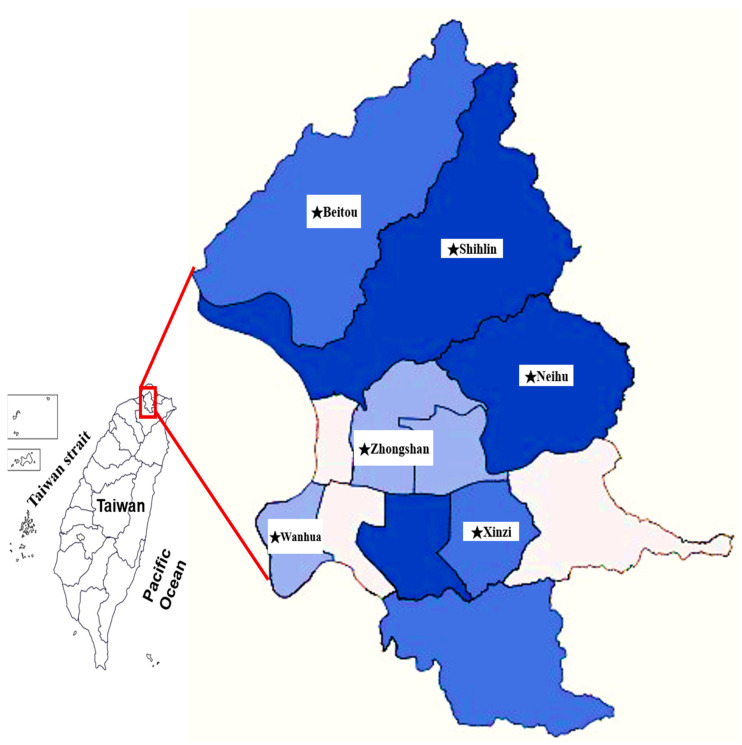
Map of Taipei city in Taiwan showing the various collection sites (indicated as ★) for ticks removed from dogs. The different shades of blue color indicate the tick collection rate (■, 20–40%; ■, 5–10%; ■, <5%).

All tick specimens were collected from May 2012 to April 2013 and were carefully removed from stray/domestic dogs from various localities in six districts of Taipei city in northern Taiwan (Figure 1). A total of 1186 ticks including 461 females, 444 males, and 281 nymphs were collected. All collected ticks were subsequently stored in separate vials filled with 75% ethanol solution, and tick species were further identified based on their morphological features [3,38]. In general, the external characteristics of the *H. hystricis* tick were photographed for species identification for ticks collected from dogs using a dissecting microscope (SMZ 1500, Nikon, Tokyo, Japan), as described previously [39].

### 2.2. Genomic DNA Extraction from Tick Specimens

Tick specimens were immersed in 75% ethanol solution and cleaned for 3–5 min by sonication. After being washed twice in sterile distilled water, the total genomic DNA was extracted from the individual tick specimen. In general, each tick specimen was homogenized with a TissueLyser II apparatus (Qiagen, Hilden, Germany) using a microcentrifuge vial containing 180 μL lysing buffer solution supplied by the commercial kit (DNeasy Blood & Tissue Kit, Qiagen, Taipei, Taiwan). After centrifugation of homogenate, the supernatant fluid was further processed by a DNeasy Blood & Tissue Kit, as instructed by the manufacturer. The eluted fluid was collected for quantifying the DNA concentration with an Epoch spectrophotometer (Biotek, Winooski, VT, USA). The ratio of absorbance at 260 nm and 280 nm was used to assess the purity of DNA, and a ratio of 1.6~1.8 was accepted for PCR analysis. The extracted DNA samples were stored at −80 °C until further assays.

### 2.3. Rickettsia Detection by Nested Polymerase Chain Reaction (PCR)

*Rickettsia* detection was performed by a nested-PCR assay using each tick extraction as a template, and two primer sets based on the citrate synthase gene (*gltA*) of *Rickettsia* were used for PCR assay. Firstly, the primer set of RpCS.877p/RpCs.1258n was used as the forward/reverse primer for amplifying the initial product of *gltA*. Afterward, the species-specific primer set of RpCS.896p/RpCS.1233n was performed for nested-PCR to amplify a *Rickettsia*-specific DNA product approximately 338 bp, as described previously [21]. The Taq polymerase and all PCR reagents were used following the instruction of the supplier (Takara Shuzo Co., Ltd., Kyoto, Japan). In general, each 25 μL reaction mixture contained 1.5 μL of forward and reverse primers, 2.5 μL of 10X PCR buffer (Mg^2+^), 2 μL of dNTP mixture (10 mM each), 3 μL of DNA template, and 1 unit of Taq DNA polymerase, and was filled up with ddH_2_O. The negative control was added with an adequate amount of sterile distilled water, and the positive control was used for our internal check. PCR amplification was conducted with a thermocycler (Veriti, Applied Biosystems, Taipei, Taiwan), and the initial PCR amplification was performed with a denaturation at 95 °C for 5 min and amplified for 35 cycles under the following conditions: denaturation at 95 °C for 30 s, annealing at 54 °C for 30 s, and extension at 72 °C for 1 min, followed by a final extension step at 72 °C for 3 min. For the nested-PCR assay, the following conditions were used: denaturation at 95 °C for 5 min, followed by amplification for 40 cycles under the same conditions as the initial PCR, except for annealing at 50 °C for 30 s. All amplified PCR products were electrophoresed on 1.5% agarose gels and then stained with ethidium bromide. Thereafter, the DNA bands on gels were observed in a UV box. Molecular size was determined by comparing with a standard marker of 100 bp DNA ladder (GeneRuler, Thermo Scientific, Taiwan). In parallel with each amplification, a negative control of distilled water was included.

### 2.4. Genetic Relatedness Determined by Phylogenetic Analysis

Each 10 μL of selected samples was submitted for DNA sequencing (Mission Biotech Co., Ltd., Taiwan). After further purification, sequencing reaction was conducted for 25 cycles (same primer set and conditions of nested-PCR assay) using the dye-deoxy terminator reaction method by the Sequencing Kit with a DNA Sequencer (ABI Prism 377, Applied Biosystems, Foster City, CA, USA). The resulting *gltA* sequences were first edited by BioEdit software (V5.3), and BLAST analysis was conducted to compare the GenBank nucleotide in NCBI, and then aligned with the CLUSTAL W software (Version 2.0) [40]. Thereafter, the aligned sequences of *Rickettsia gltA* genes from 13 Taiwan specimens were compared with other 16 *Rickettsia* sequences documented in GenBank (Table 1). Phylogenetic analysis was completed by using the neighbor-joining (NJ) and maximum likelihood (ML) methods to estimate the entire alignment using MEGA X software [41]. The intra- and inter-species variations in genetic distance values were analyzed by the Kimura two-parameter model [42]. The reliability of the phylogenetic trees was analyzed with 1000 bootstrap replications [43].

### 2.5. GenBank Accession Numbers of Submitted Nucleotide Sequences

The nucleotide sequences of the *gltA* genes of 13 *Rickettsia* strains identified from the *H. hystricis* ticks of Taiwan were assigned with the GenBank accession numbers (PQ212712-24) indicated in Table 1. For phylogenetic analysis, the nucleotide sequences of *gltA* genes from other 16 *Rickettsia* strains documented in GenBank were also included for comparison (Table 1).

### 2.6. Statistical Analysis

The percentage of *Rickettsia* infection between the stray dogs and domestic dogs was compared and analyzed by Pearson’s chi-square test.

## 3. Results

### 3.1. Molecular Detection of Rickettsia Infection in H. hystricis Ticks of Taiwan

**Figure 2:**
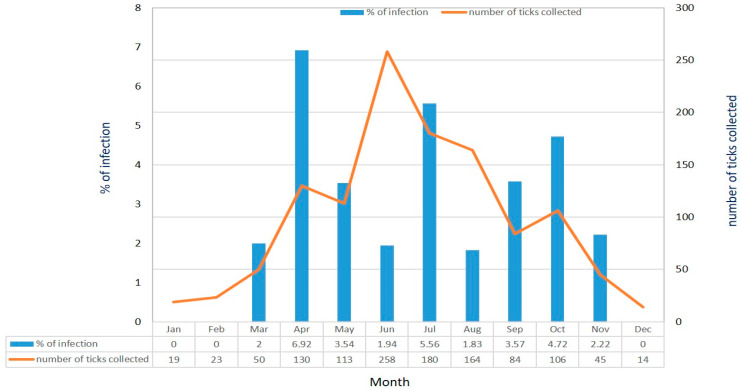
Monthly prevalence of *Rickettsia* infection and number of *H. hystricis* ticks collected from dogs (May 2012–April 2013) in Taipei city of Taiwan.

*Rickettsia* infection was detected in *H. hystricis* ticks by nested-PCR assay targeting the *gltA* gene. In general, *Rickettsia* infection was detected in 3.46% (41/1186) of *H. hystricis* ticks, and it was detected in females, males, and nymphs of *H. hystricis* ticks with infection rates of 3.2% (13/461), 3.6% (16/444), and 4.27% (12/281), respectively (Table 2). The prevalence of *Rickettsia* infection in *H. hystricis* ticks infesting stray dogs (4.15%) is significantly higher than ticks infesting domestic dogs (1.11%) (Table 2). In addition, the monthly prevalence of *Rickettsia* infection was observed from March to November, and the highest infection rate was detected in April (6.92%) followed by a higher infection rate in July (5.56%), October (4.72%), September (3.57%), and May (3.54%) (Figure 2).

### 3.2. Genetic Relatedness of Rickettsia *spp.* Detected in H. hystricis Ticks

In this study, the aligned sequences of *gltA* gene fragments from 13 *Rickettsia* specimens of Taiwan were compared with the downloaded sequences of 16 other *Rickettsia* strains documented in GenBank (Table 1). Our results reveal that 11 *Rickettsia* strains detected in *H. hystricis* ticks of Taiwan were genetically affiliated with the genospecies of *R. felis* with a high sequence similarity of 99.7–100%, and one *Rickettsia* strain was affiliated with the genospecies of *R. aeschlimannii* and *R. raoultii* with sequence similarities of 100% and 99.7%, respectively (Table 3). Based on the genetic distance (GD) values of the *gltA* gene, the intra- and inter-species analysis revealed lower levels (GD < 0.003, <0.0, and <0.003) of genetic divergence within the *Rickettsia* strains of Taiwan, as compared with the type strain of *R. felis*, *R. aeschlimannii*, and *R. raoultii*, respectively (Table 3). However, a higher level (GD > 0.038) of genetic divergence was observed compared to other *Rickettsia* strains.

### 3.3. Phylogenetic Analysis of Rickettsia *spp.* Detected in H. hystricis Ticks

**Figure 3:**
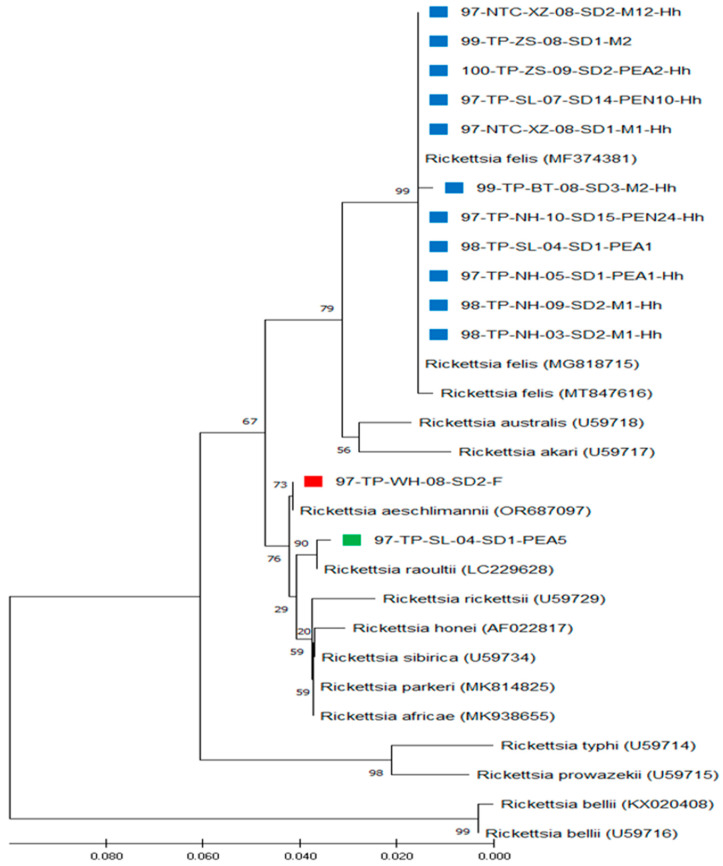
Genospecies identification based on the citrate synthase gene (*gltA*) sequences of *Rickettsia* among 13 specimens identified from *Haemaphysalis hystricis* ticks of Taiwan and 16 other *Rickettsia* strains validated in GenBank. The Taiwan strains were affiliated with the genospecies of *R. felis* (indicated as ■), *R. aeschlimannii* (indicated as ■), and *R. raoultii* (indicated as ■). The phylogenetic tree was constructed by the maximum likelihood (ML) method and analyzed with 1000 bootstrap replicates. Numbers at the nodes represent the reliability of each branch in the tree. Branch length is drawn proportional to the estimated sequence divergence.

Based on the aligned sequences of *gltA* genes among 13 Taiwan strains and 16 other *Rickettsia* strains, phylogenetic trees were constructed by neighbor-joining (NJ) and maximum likelihood (ML) methods. The results demonstrate congruent basal topologies with eight major clades of *Rickettsia* that can be easily distinguished by ML analysis (Figure 3), which were also supported by NJ analysis (Supplementary Figure S1). Briefly, 11 *Rickettsia* strains from Taiwan constitute a monophyletic clade genetically affiliated with the genospecies of *R. felis*, and one *Rickettsia* strain of Taiwan was genetically affiliated with the genospecies of *R. aeschlimannii* and *R. raoultii*, respectively (Figure 3). Our results reveal a lower genetic divergence within the same genospecies of *Rickettsia* detected in *H. hystricis* ticks from Taiwan, but higher genetic variation among other *Rickettsia* groups from different biological and geographical origins.

## 4. Discussion

Our investigation provides the first molecular survey and genetic identification of *Rickettsia* species detected in *H. hystricis* ticks of Taiwan. In general, the *Rickettsia* species detected in *H. hystricis* ticks are genetically affiliated with the genospecies of *R. felis*, *R. aeschlimannii*, and *R. raoultii* (Figure 3 and Figure S1, which are different from other tick-borne pathogens discovered in previous reports described in Malaysia [9,10], China, [5] and Taiwan [4,11]. Although *R. felis*, *R. rhipicephali*, and *R. massiliae* have been detected in *Ixodes granulatus* and *Rhipicephalus haemaphysaloides* ticks from Taiwan [44,45,46], this study described the initial detection of *R. aeschlimannii* and *R. raoultii* in *H. hystricis* ticks of Taiwan. In previous reports, *R. aeschlimannii* has been recognized as a human pathogen found in tourists returning from Morocco and South Africa, and identified from *Hyalomma marginatus* and *Rhipicephalus appendiculatus* ticks [47,48]. Thus, this study reveals the first confirmed evidence regarding the existence of various *Rickettsia* species in *H. hystricis* ticks of Taiwan.

The genetic identity of *Rickettsia* spp. detected in *H. hystricis* ticks can be determined by the phylogenetic analysis of genetic relatedness among *Rickettsia*. In previous studies, sequence analysis based on the *gltA* gene of *Rickettsia* strains identified from different origins has been proved as a feasible method for determining the genetic identity of *Rickettsia* detected in various geographical and biological sources [13,34,35,36,37,44,45,46]. In the present study, sequence similarity based on the *gltA* gene of *Rickettsia* strains detected in *H. hystricis* ticks of Taiwan revealed a highly genetic homogeneity affiliated with the genospecies of *R. felis* (99.7–100% similarity), *R. aeschlimannii* (100% similarity), and *R. raoultii* (99.7%) (Figure 3, Table 3). The *R. felis* strains are mainly affiliated with the *Rickettsia* strain identified in the *Rhipicephalus sanguineus* tick of Taiwan (GenBank accession no. MT847616). However, *R. aeschlimannii* and *R. raoultii* are mainly affiliated with the *Rickettsia* strains identified in the *Dermacentor reticulatus* tick of Russia (GenBank accession no. OR687097) and the *Hyalomma marginatum* tick of Portugal (GenBank accession no. LC229628), respectively. The phylogenetic analysis constructed by both NJ and ML trees strongly supports the distinction between *Rickettsia* strains in *H. hystricis* ticks of Taiwan and other *Rickettsia* genospecies identified from various geographic and biological sources. Thus, our findings reveal the first evidence for the genetic identity of *Rickettsia* strains detected in *H. hystricis* ticks of Taiwan.

The factors accounting for the monthly prevalence of *Rickettsia* infection in *H. hystricis* ticks of Taiwan remain unclear. In this study, *Rickettsia* infection in *H. hystricis* ticks seems highly associated with the seasonal abundance of the tick population. Indeed, the adult ticks were mostly collected from the infested dogs during early spring (March) to late autumn (November) and *Rickettsia* infection was detected during these months (Figure 2). In addition, a significant difference in Rickettsia infection in *H. hystricis* ticks was observed between stray (4.15%) and domestic (1.11%) dogs (Table 2). A possible factor responsible for this variation is the total number of collected ticks from stray/domestic (915/271) dogs. Indeed, stray dogs tend to live in groups, and they usually have 5–7 dogs in a single herd, which provides an ideal contact situation for cross-infection of *Rickettsia* pathogens. Another possible factor described by the previous study indicates that antibiotic treatment reduces the *Rickettsia* and *Coxiella* endosymbionts within ticks [49]. In Taiwan, domestic dogs usually have routine vaccination and various treatments, including antibiotic therapy, and this process may also reduce *Rickettsia* infection in domestic dogs. Thus, there is an essential need for developing control strategies for reducing the population of stray dogs in Taiwan.

The actual mechanism responsible for the transmission of the *Rickettsia* pathogen in *H. hystricis* ticks remains elusive. In this study, our results revealed a higher prevalence of *Rickettsia* infection in male *H. hystricis* ticks, and this high prevalence of *Rickettsia* infection in male ticks may have been inherited from infected nymphs (Table 2). Indeed, previous reports have verified that *Rickettsia* pathogens can be preserved transstadially/transovarialy within vector ticks [19,20,50]. The co-feeding mechanism may also contribute to another possible mode of transmission, in which ticks feeding in close proximity to another infected tick on the same host may facilitate *Rickettsia* transmission between these ticks [51,52]. Because of the close contact between dogs and humans, our results may highlight the role of dogs serving as carrier/reservoir hosts for *Rickettsia* transmission in nature. Nevertheless, further studies focusing on vector competence and seasonal variation in *H. hystricis* ticks for various tick-borne pathogens would help to illustrate their epidemiological significance for human infection in Taiwan.

## 5. Conclusions

This study conducts the first molecular screening and genetic characterization of the *Rickettsia* agents present in *H. hystricis* ticks infesting dogs of Taiwan. The genetic relatedness based on the phylogenetic analyses of the *gltA* gene reveals its affiliation with the genospecies of *R. felis*, *R. aeschlimannii*, and *R. raoultii*. The human pathogenic strain of *R. aeschlimannii* was firstly discovered in *H. hystricis* ticks. Because dogs serve as companion animals to humans, this discovery of various *Rickettsia* agents in *H. hystricis* ticks may highlight the potential threat for human infections in Taiwan. Further studies for reducing the population of stray dogs in Taiwan as well as the preventive treatment with acaricide for domestic dogs will facilitate the control of ticks and tick-borne pathogens in Taiwan.

## Figures and Tables

**Table 1:** *Rickettsia* strains from Taiwan compared with other *Rickettsia* species documented in GenBank.

Strain/Species	Origin of *Rickettsia* Strain		*gltA* Gene Accession Number ^a^
	Biological	Geographic	
Taiwan strain			
97-TP-SL-04-SD1-PEA5	*Haemaphysalis hystricis*	Taiwan	**PQ212712**
97-TP-WH-08-SD2-F	*Haemaphysalis hystricis*	Taiwan	**PQ212713**
97-NTC-XZ-08-SD1-M1-Hh	*Haemaphysalis hystricis*	Taiwan	**PQ212714**
97-NTC-XZ-08-SD2-M12-Hh	*Haemaphysalis hystricis*	Taiwan	**PQ212715**
97-TP-NH-05-SD1-PEA1-Hh	*Haemaphysalis hystricis*	Taiwan	**PQ212716**
97-TP-NH-10-SD15-PEN24-Hh	*Haemaphysalis hystricis*	Taiwan	**PQ212717**
97-TP-SL-07-SD14-PEN10-Hh	*Haemaphysalis hystricis*	Taiwan	**PQ212718**
98-TP-NH-03-SD2-M1-Hh	*Haemaphysalis hystricis*	Taiwan	**PQ212719**
98-TP-NH-09-SD2-M1-Hh	*Haemaphysalis hystricis*	Taiwan	**PQ212720**
98-TP-SL-04-SD1-PEA1	*Haemaphysalis hystricis*	Taiwan	**PQ212721**
99-TP-BT-08-SD3-M2-Hh	*Haemaphysalis hystricis*	Taiwan	**PQ212722**
99-TP-ZS-08-SD1-M2	*Haemaphysalis hystricis*	Taiwan	**PQ212723**
100-TP-ZS-09-SD2-PEA2-Hh	*Haemaphysalis hystricis*	Taiwan	**PQ212724**
*Rickettsia felis*	*Ctenocephalides felis*	Austria	MF374381
*Rickettsia felis*	Booklice from herbals	China	MG818715
*Rickettsia felis*	*Rhipicephalus sanguineus*	Taiwan	MT847616
*Rickettsia australis*	Human	Australia	U59718
*Rickettsia akari*	Human	USA	U59717
*Rickettsia aeschlimannii*	*Dermacentor reticulatus*	Russia	OR687097
*Rickettsia raoultii*	*Hyalomma marginatum*	Portugal	LC229628
*Rickettsia rickettsii*	*Dermacentor andersoni*	USA	U59729
*Rickettsia parkeri*	*Amblyomma ovale*	Mexico	MK814825
*Rickettsia africae*	Diatom	Italy	MK938655
*Rickettsia honei*	Human	Australia	AF022817
*Rickettsia sibirica*	*Dermacentor nuttalli*	USSR	U59734
*Rickettsia typhi*	Human	USA	U59714
*Rickettsia prowazeki*	Human	Poland	U59715
*Rickettsia bellii*	*Amblyomma pseudoconcolor*	Brazil	KX020408
*Rickettsia bellii*	*Dermacentor variabilis*	USA	U59716

**^a^** Bold accession numbers of GenBank were submitted by this study.

**Table 2:** Molecular detection of *Rickettsia* infection in *Haemaphysalis hystricis* ticks infested stray/domestic dogs by nested-PCR assay targeting the *gltA* gene of the *Rickettsia* pathogen.

Parasitized Host	*Rickettsia* spp. Detected in Various Life Stages of Tick			Total
	Partially Engorged Female P/E ^a^ (%)	Flat Male P/E ^a^ (%)	Partially Engorged Nymph P/E ^a^ (%)	No. Positive/ No. Examined (%)
Stray dogs	11/302 (3.64)	15/358 (4.19)	12/255 (4.71)	38/915 (4.15) b
Domestic dogs	2/159 (1.26)	1/86 (1.16)	0/26 (0)	3/271 (1.11) b
Total (%)	13/461 (3.2)	16/444 (3.6)	12/281 (4.27)	41/1186 (3.46)

**^a^** P/E = No. of positive tick/no. of tick examined; ^b^ chi-square test, *p* = 0.015.

**Table 3:** Intra- and inter-group analysis of genetic distance values ^a^ based on the *gltA* gene sequences between the *Rickettsia* strains from Taiwan and other *Rickettsia strains* documented in GenBank.

*Rickettsia* Strains ^b^	1	2	3	4	5	6	7	8	9	10	11	12	13	14	15
1. *Rickettsia felis* (MF374381)	—														
2. 100-TP-ZS-09-SD2-PEA2-Hh (Taiwan)	0.000	—													
3. 97-NTC-XZ-08-SD2-M12-Hh (Taiwan)	0.000	0.000	—												
4. 98-TP-SL-04-SD1-PEA1 (Taiwan)	0.000	0.000	0.000	—											
5. 98-TP-NH-09-SD2-M1-Hh (Taiwan)	0.000	0.000	0.000	0.000	—										
6. 99-TP-BT-08-SD3-M2-Hh (Taiwan)	0.003	0.003	0.003	0.003	0.003	—									
7. *Rickettsia aeschlimannii* (OR687097)	0.038	0.038	0.038	0.039	0.039	0.042	—								
8. 97-TP-WH-08-SD2-F (Taiwan)	0.038	0.038	0.038	0.039	0.039	0.042	0.000	—							
9. *Rickettsia raoultii* (LC229628)	0.038	0.038	0.038	0.039	0.039	0.042	0.006	0.006	—						
10. 97-TP-SL-04-SD1-PEA5 (Taiwan)	0.041	0.042	0.042	0.042	0.042	0.046	0.010	0.010	0.003	—					
11. *Rickettsia parkeri* (MK814825)	0.041	0.042	0.042	0.042	0.042	0.046	0.003	0.003	0.003	0.007	—				
12. *Rickettsia sibirica* (U59734)	0.041	0.042	0.042	0.042	0.042	0.046	0.003	0.003	0.003	0.007	0.000	—			
13. *Rickettsia rickettsii* (U59729)	0.053	0.053	0.053	0.054	0.054	0.057	0.017	0.017	0.017	0.020	0.013	0.013	—		
14. *Rickettsia typhi* (U59714)	0.089	0.090	0.090	0.091	0.091	0.094	0.082	0.082	0.082	0.086	0.086	0.086	0.090	—	
15. *Rickettsia bellii* (KX020408)	0.194	0.196	0.196	0.193	0.193	0.197	0.168	0.168	0.168	0.173	0.173	0.173	0.169	0.213	—

^a^ The pairwise distance calculation was performed by the method of Kimura 2-parameter, as implemented in MEGA X (Kumar et al., 2018 [41]). ^b^ Strains 1, 7, 9, and 11–15 indicate the various *Rickettsia* species documented in GenBank.

## Data Availability

The GenBank accession numbers (PQ212712-24) submitted by this study are available in GenBank after publication.

## Supplementary Materials

The following supporting information can be downloaded at: https://www.mdpi.com/article/10.3390/microorganisms13020424/s1.
