# Supplementary material for: First Molecular Survey and Genetic Characterization of Rickettsia spp. in Haemaphysalis hystricis Ticks Infesting Dogs in Taiwan
Source: Microorganisms. 2025 Feb 15;13(2):424. doi: 10.3390/microorganisms13020424 (PMC11857873; doi:10.3390/microorganisms13020424)
Supplement: Supplementary file 1 [file microorganisms-13-00424-s001.zip › microorganisms-3458118-supplementary.pdf]

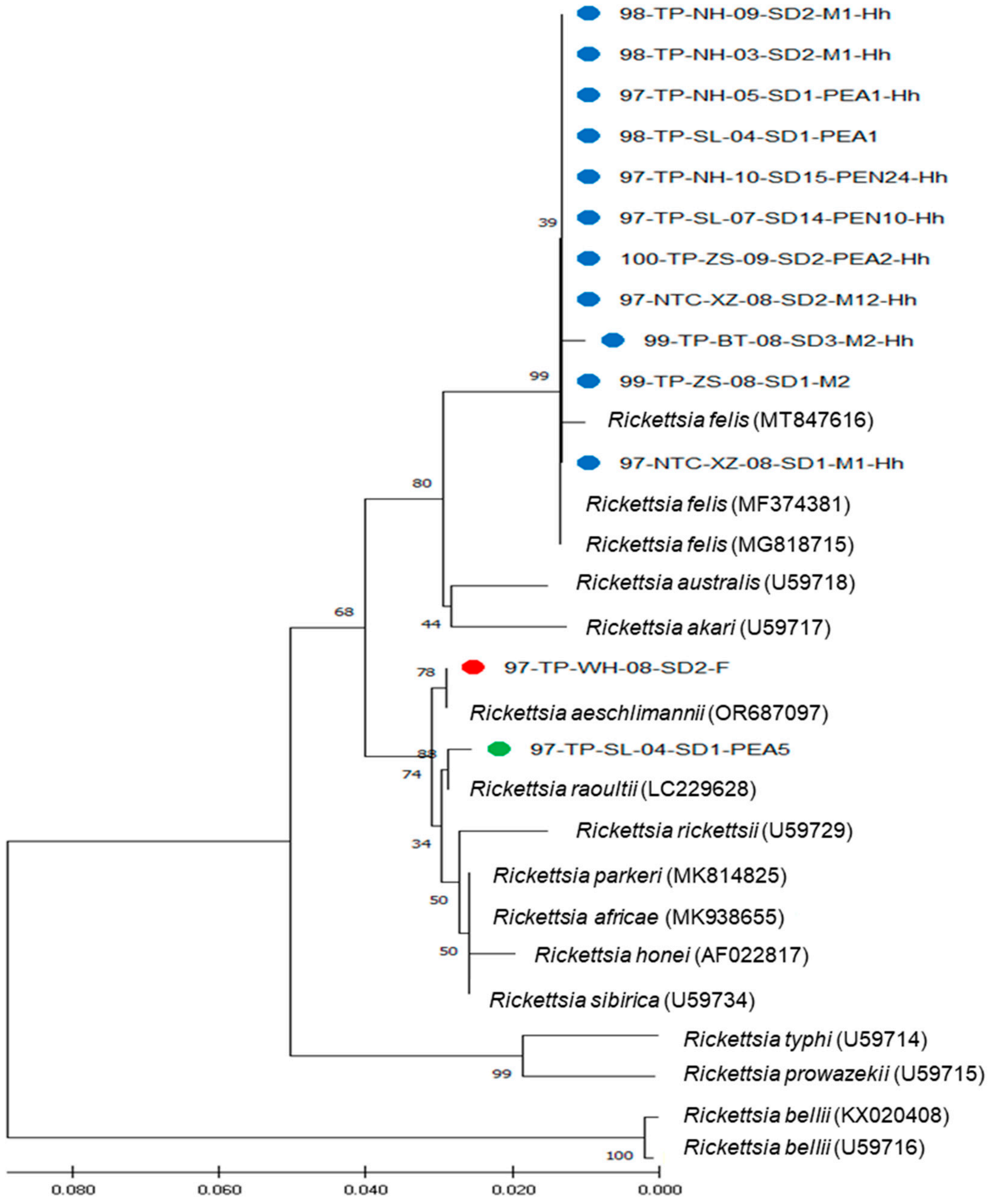

Supplementary Figure S1. Genospecies identification based on the citrate synthase gene (*gltA*) sequences of *Rickettsia* between 13 specimens identified from *Haemaphysalis hystricis* ticks of Taiwan and 16 other *Rickettsia* strains validated in GenBank. The Taiwan strains were affiliated to the genospecies of *R. felis* (indicated as ●), *R. aeschlimannii* (indicated as ●) and *R. raoultii* (indicated as ●), respectively. The phylogenetic tree was constructed by neighbour-joining (NJ) method and analyzed with 1000 bootstraps replicates. Numbers at the nodes represent the reliability of each branch in the tree. Branch length is drawn proportional to the estimated sequence divergence.
